# Inadequate treatment in internships: a comparison between medical and other students 

**DOI:** 10.3205/zma001441

**Published:** 2021-02-15

**Authors:** Sonia Bormuth, Hanns Ackermann, Johannes Schulze

**Affiliations:** 1 Goethe-University Frankfurt/Main, Institute of Occupational, Social and Environmental Medicine, Frankfurt/Main, Germany; 2 Goethe-University Frankfurt/Main, Institute of Biostatistics and Mathematical Modelling, Frankfurt/Main, Germany

**Keywords:** aggression, communication, stress, psychological, harassment, non-sexual, bullying, comparative study

## Abstract

**Aims: **Inadequate treatment is one of the factors interfering with a successful social and working life. Among students, it can impair their health and learning progress. In the field of medicine the problem of inadequate treatment seems widespread. This study examines wether inadequate treatment in internships differs between medicine and other academic disciplines.

**Method: **Using a questionnaire, the frequency, forms and severity of inadequate treatment among students were compared between the disciplines of medicine, civil engineering and teaching.

**Results: **69,3% of medical students reported inadequate treatment during their internships, about twice as many as students of other disciplines. The ratios of verbal, non-verbal and organisational inadequate treatment were similar between the different academic disciplines. However, medical students executed tasks without receiving sufficient safety precautions or training significantly more often (sevenfold) than students of other disciplines. In total however, the experienced incidents of inadequate treatment were seen as similarly severe across the different academic fields.

**Conclusion:** Inadequate treatment of students during internships is a larger problem in medicine than in civil engineering or teaching, particularly concerning the performance of unsafe tasks. With regard to the health of students and patients, inadequate treatment in the medical education should be tackled. Previous studies suggest that this goal can be achieved only through longtime extensive measures on the level of students, lecturers, faculty and teaching hospitals.

## Zusammenfassung

**Zielsetzung: **Unangemessene Behandlung gehört zu den Faktoren, die ein erfolgreiches Zusammenleben und -Arbeiten erschweren und bei Studierenden Gesundheit und Lernerfolg beeinträchigen. In der Medizin scheint das Problem unangemessener Behandlung weit verbreitet. Diese Studie untersucht, ob unangemessene Behandlung in Praktika sich zwischen Medizin- und anderen Studierenden unterscheidet.

**Methodik: **Anhand eines Fragebogens werden die Häufigkeit, Art und Weise sowie Schwere von Vorfällen unangemessener Behandlung zwischen den Studiengängen Humanmedizin, Bauingenieurwesen und Lehramt verglichen.

**Ergebnisse:** Medizinstudierende geben mit 69,3% etwa doppelt so häufig unangemessene Behandlung an wie Studierende anderer Fächer. Dabei sind die Anteile verbaler, nonverbaler und organisatorischer unangemessener Behandlung zwischen den Studiengängen ähnlich verteilt, jedoch ist der Anteil an Medizinstudierenden, die Aufgaben ohne ausreichende Sicherheitsvorkehrungen oder Einweisung ausgeführt haben, sehr viel höher (7x) als in den anderen Studiengängen. Die erlebten Vorfälle von unangemessener Behandlung werden in den verschiedenen Studiengängen im Mittel als gleich schwerwiegend angesehen.

**Schlussfolgerung: **Die unangemessene Behandlung von Studierenden während Praktika ist in der Medizin ein größeres Problem als in den Berufen Bauingenieurwesen und Lehramt, insbesondere auch in Bezug auf das Ausüben unsicherer Tätigkeiten. Mit Hinblick auf die Gesundheit von Studierenden und Patienten sollte gegen unangemessene Behandlung in der medizinsichen Ausbildung vorgegangen werden. Bisherige Studien lassen darauf schließen, dass dies nur durch langjährige umfangreiche Maßnahmen auf Ebene der Studierenden, der Lehrenden und der Fakultät bzw. Lehrkrankenhäuser gelingen kann.

## 1. Introduction

Demeaning or lacking communication is an elementary part in most forms of inadequate treatment (IAT). This may happen in a verbal, nonverbal or organisational manner [1], [2], [3], [4]. IAT is seen in education and university studies mainly in the context of internship rotations [5], in which students experience an essential part of their occupational imprinting by the temporary transfer from a learning environment into the working conditions of their desired occupation in the framwork of a “hidden curriculum” [6], [7], [8]; in medical students this transition will form their perception of the medical culture and their sense of appropriate conduct [9], [10], [11], [12]. 

Experiencing IAT during their training can have expensive ramifications with negative consequences for their own psychological well being [13], [14], [15], [16], the medical self-conception [17], [18] as well as their attitude toward patients [17], [19] and further generations of students [2], [6], [8]. 

Consequently, IAT is contrary to the intentions of the German legal framework for medical studies (Approbationsordnung) [https://www.gesetze-im-internet.de/_appro_2002/BJNR240500002.html] as well as the professional framework for physicians [20] which intend to educate and and upskill physicians to work responsibly, competently, loyally, ethically and patient centered. 

It is assumed that a steep hierarchy in medicine [21], [22], [23], lack of time and stress with patient treatment are causing IAT [24], [25], [26], [27], as well as the professional socialisation forwarded from generation to generation as “hidden curriculum” in a system promoting IAT [2], [10], [11]. 

IAT as a phenomenon in medical education has been investigated in a number of studies sind the 1980‘s, some of those are compiled in table 1 (Tab. 1). However, comparing data across countries and languages is difficult expecially since some of the terms used to characterize IAT do not have an identical translation. Gagyor et al. already had to use interpretations of the english terms “abuse”, “mistreatment” and “harassment” in their German language questionnaire for “inadequate treatment” [28]. 

Most studies consider IAT as a problem specific to medicine and medical education, without considering that IAT is pervasive in many different occupations [1], [2], [3], [29] as research in mobbing suggests [30]. Consequently nearly no studies exist to compare the incidence of IAT across different university studies. However, this appears to be necessary and relevant to better understand IAT as a specific or nonspecific phenomenon and develop targeted solutions.

In this paper we investigate whether IAT of students in medical internships is different from IAT in internships in other occupations, and what characterizes these differences. We define IAT as “behaviour which is felt as inadequate or disapproved in its characteristic or its extent by at least one of the people present in the situation, and has thus provoked feelings of surprise, anger, shame or disappoiontment”. Specifically we have investigated whether IAT in medical internships is equally present in frequency, manner and severity compared to other university studies.

We compared medical students with civil engineer students (civ eng) and students of high school teaching degrees (TD). Each degree program qualifies for a clearly defined job description and requires at least one mandatory preparative internship of 12 to 15 weeks duration; this internship also requires the students to work independently under supervision.

## 2. Methods

### Study design

After a literature review about IAT, 5 semi structured interviews were conducted in each degree program; based on these interviews and the publication of Gagyor et al. of negative experiences in medical students [5] an a-version of a questionnaire was compiled. This preliminary version included demografic data (sex, age, ethnic background, study semester and intended occupation), personal experience and appraisal of IAT as well as grading typical situations. Ambiguous phrases were corrected and duplicates were removed resulting in the final form of the questionnaire in its German version (see attachment 1 ). In order to answer the main question this manuscript mostly represents the questionnaire part concerning IAT experience of students during internships. 

To better compare different study courses a definition of inadequate treatment was given for all participants in an introduction to the questionnaire (see introduction). In addition, course specific phrases were given in general terms if applicable (e.g. “superior” instead of “doctor” or “teacher”). 

#### Data collection

For all three study courses the questionnaire was distributed to all participants in a mandatory course during the winter term 2017/2018; participation was voluntary for all students. For medical studies 5^th^ term students of the medical study program at the Goethe-University Frankfurt/Main were surveyed in the mandatory “seminar in theoretical pathophysiology and pharmacology”. Bachelor degree students in civil engineering were surveyed at the Technical University Darmstadt, also during their 5^th^ term, in the lecture “Geotechnique I”. 

Teaching degree students were surveyed at the Goethe-University Frankfurt/Main during their 3rd or 4th term in the background seminar parallel to their internship semester. In contrast to the procedure in the other two groups the questionnaire was distributed during one seminar and completed at home, not during the seminar. 

#### Data evaluation

After generating a database the statistical package BiAS [https://www.bias-online.de/index.html] from the department of statistics at the institute of biostatistics and mathematic modelling, Goethe-University Frankfurt/Main, Germany, was used to calculate parameters of inferential statistics. 

Group differences were tested with Fisher’s exact test (effect size Cohen‘s ω; test of Fisher, Freeman and Halton), the Wilcoxon Mann Whitney U-test (effect size Rosenthal’s R) and the Kruskal-Wallis test (effect size η^2^). If a significant difference was found with the Kruskal-Wallis test (p<0,05) with at least an intermediate effect size, the test was supplemented with multiple Dunn pairwise comparisons with significance thresholds adapted by the approximation of Bonferroni and Holm. Differences were considered relevant if significant (p<0,05) differences were observed with effect sizes ω≥0,3, R≥0,3 and/or η^2^≥0,06.

This pilot study was approved by the ethics commission of the Goethe-University Frankfurt/Main, Germany.

## 3. Results

The questionnaire return rate of complete forms was 86% with medical students (261/305), 88% with civil engineering students (146/170) und 55% with teaching degree students (87/159). Demographic characteristics are compiled in Table S2 in the attachment 2 . 

Relevant group differences in the classification of potential IAT situations were seen only in three of 14 examples: Teaching degree students considered the invitation to a drink by a superior as inadequate more often than their peers in medicine or civil engineering (question 18). In questions 21 and 22 medical students considered introducing themselves loudly when entering a room (q. 21) or asking for assistance in a specific task (q. 22) more often as appropriate. 

### Frequency of IAT experiences

This parameter was asked in question 5. Of all participating medical students 69% reported experiences of IAT – more than twice as many as students from the other two study programs (civ eng 32%, TD 33%; p<0,0001, ω=0,37). 

**Figure 1:**
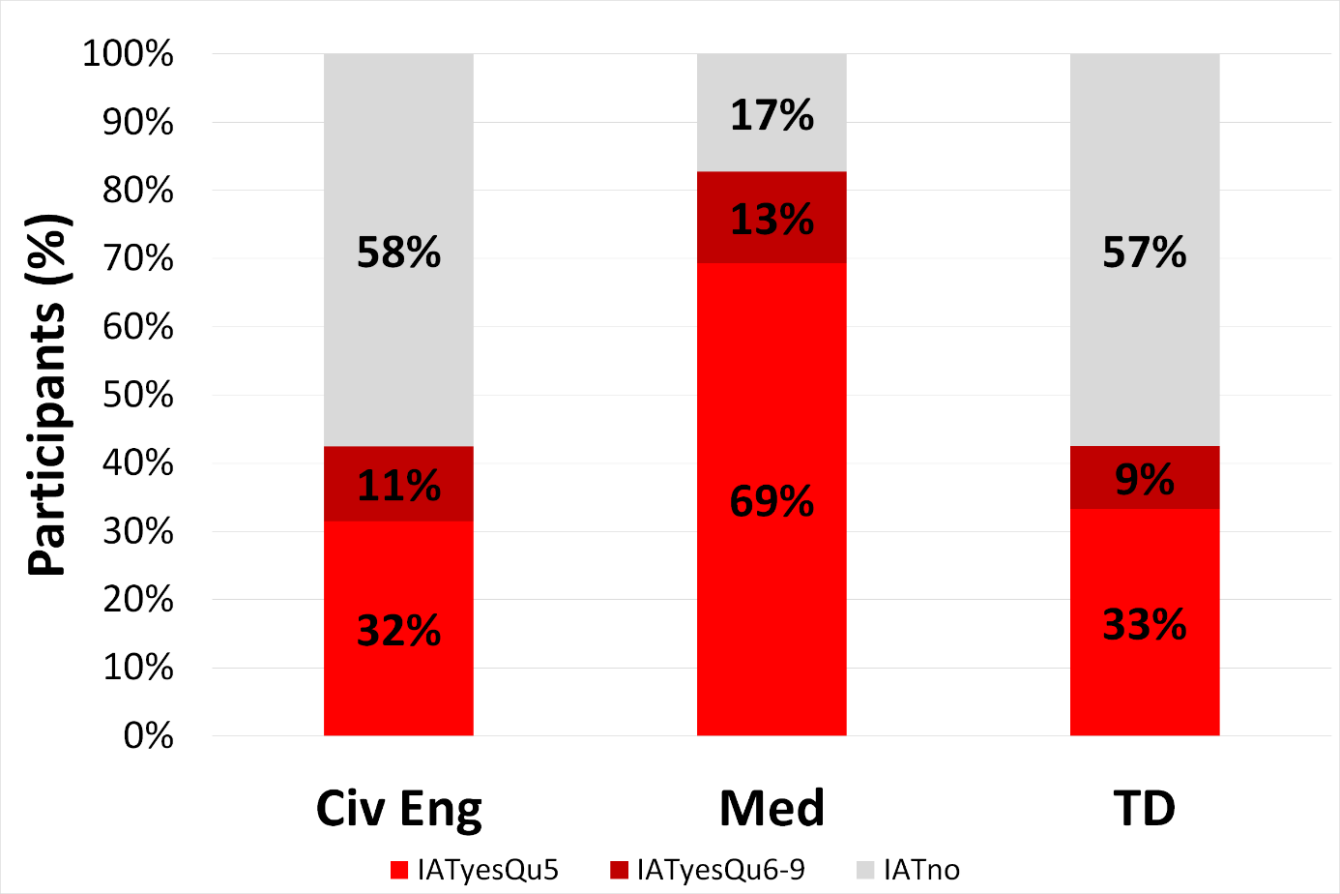
Frequency of IAT experience in the three cohors. IATyesQu5: reported overall IAT in question 5, IATyesQu6-9: additionally reported specific IAT in questions 6 to 9; IATno: no IAT experience in questions 5 to 9.

Some students who did not report IAT in question 5 listed instances of specific IAT experiences inquired in questions 6 to 9. As these students were added to the 69% reported overall IAT experiences the rate of medical students with IAT rose to 83%. Using this modified calculation for all three cohorts still resulted in roughly twice as many IAT encounters in medical students (civ eng 43%, TD 43%; p<0,0001, ω=0,42; see figure 1 (Fig. 1), table S3 in the attachment 2 ). 

#### Types of IAT experiences

**Figure 2:**
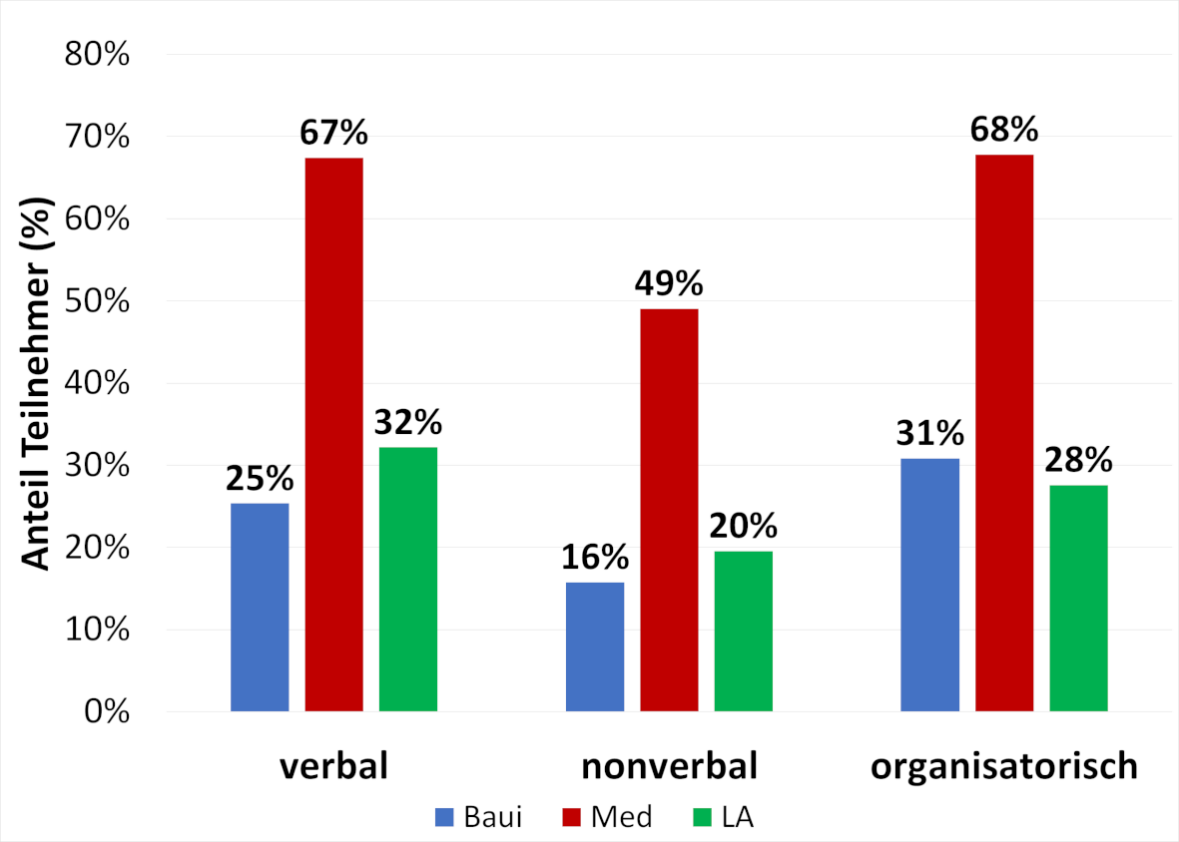
Frequency of IAT experiences in the three study programs; Values are based on all participants = 100%. Positive answers in question 9 were added to verbal IAT, mentioning in questions 6 to 8 were added to the category the participants gave in their report of the IAT. Multiple answers result in a total of >100%.

**Figure 3:**
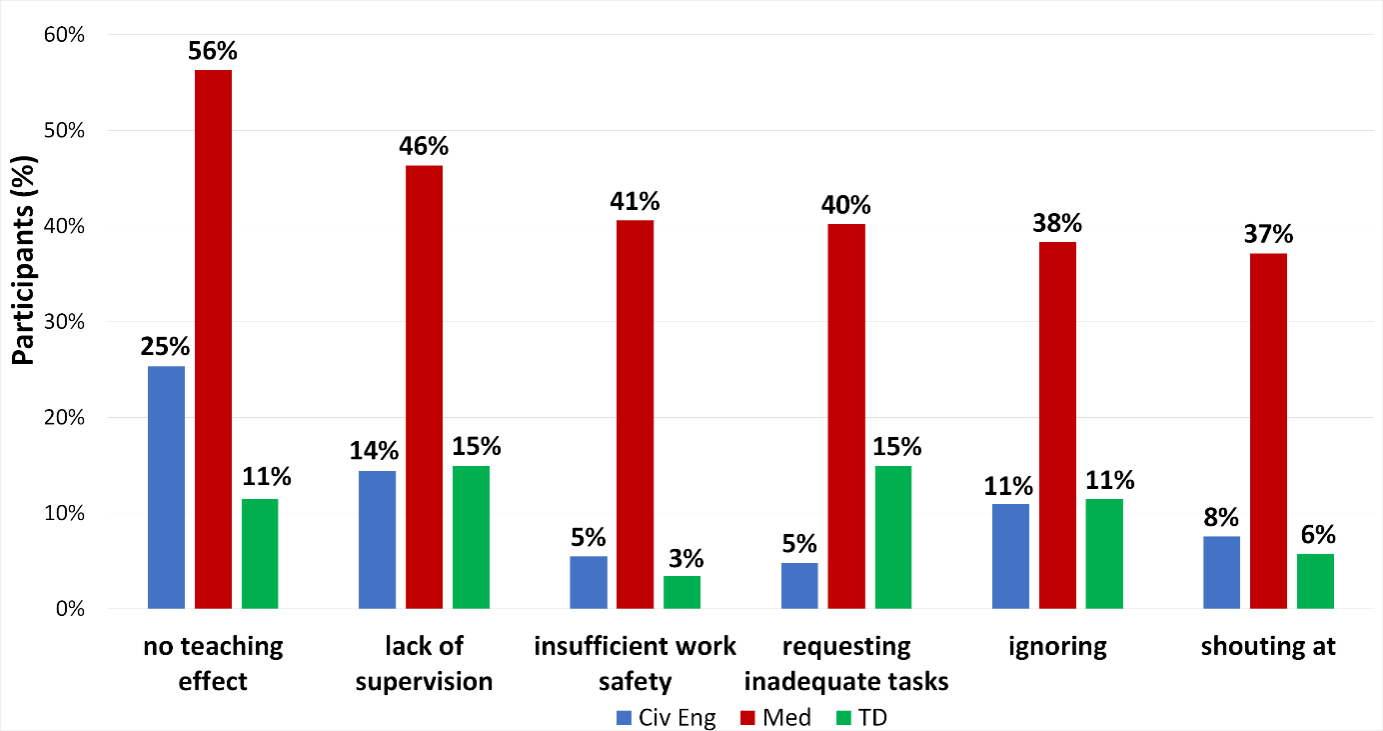
Comparison of the most frequent types of IAT in the three study programs. Values are based on all participants = 100%. Multiple answers result in a total >100%.

Figure 2 (Fig. 2) and figure 3 (Fig. 3) compare the frequencies of different IAT categories or of the 6 types of IAT most often reported by medical students. The figures include both general IAT experience from question 5 as well as specific IAT types listed in questions 6 to 9. The complete data set for the frequency of all IAT categories and types are listed in the attachment 2 in figure S4 and Tables S5 and S6.

Students in all three programs listed verbal and organisational IAT with comparable frequency, with nonverbal IAT being less frequent (see figure 2 (Fig. 2)). Also, in all three programs “no teaching result”, “insufficient supervision” and “non-observance” were mentioned among the six types of IAT most frequently experienced (see figure 3 (Fig. 3)). In all these three types miscommunication by the supervisors is possible. Direct verbal IAT like “being shouted at” were rarely listed by civil engineering students (8%) and teaching degree students (6%) but by 37% of medical students. Only medical students frequently listed lack of safety standards (41%), students of civil engineering (3%) or teaching degree (5%) mentioned it rarely. Similarly, requesting activities not permitted for students were frequently reported only in medicine with 40% (civ eng 5%, TD 15%). In all six types of IAT presented in figure 3 (Fig. 3) medical students reported IAT more frequently than students in the other programs (p and ω values are given in table S7 in attachment 2 ). 

#### Severity of IAT

18.4% of medical students and 27.3% of civil engineering students listed “tasks without a learning effect” as most severe form of IAT (answers to question 5.5), whereas teaching degree students listed “inadequate supervision” as the most severe form of IAT (20,8%). Active verbal demeanor, especially “being shouted at” or “negative statements about professional topics” were reported disproportionally often as “most severe incident” in all three study programs.

**Figure 4:**
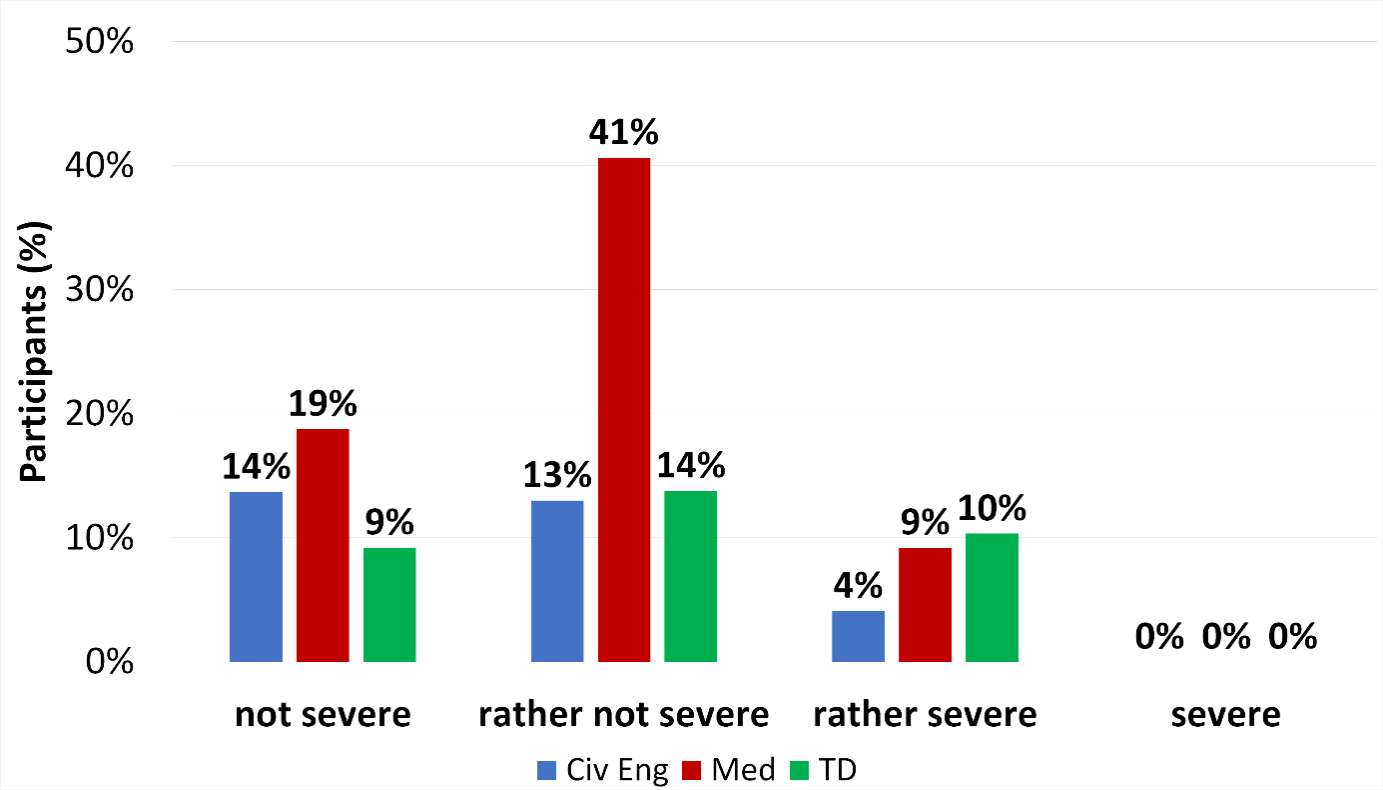
Frequency of mild and severe IAT in the incident considered the most grave during the internship. All participants=100%

Figure 4 (Fig. 4) compares the severitiy of the gravest incident of IAT yet experienced among the participants in all three study programs (answers to question 5.6). IAT graded as “rather not severe” as worst IAT yet was given by medical students more often than other students (p<0,0001, ω=0,30). Study programs were not relevantly different in the grading of IAT incidents as “not severe”, “rather not severe”, “rather severe” and “severe” by students of all three study programs (table S8 in attachment 2 ). It also is worth mentioning that no participant in any study program expereienced a severe incident of IAT.

#### Subgroup analysis

Only small, nonrelevant differences were found when comparing male and female students (table S9 in the attachment 3 ). Other subgroup analysis, e.g. in students with or without ethnic background differences were not evaluable due to small group sizes. 

## 4. Discussion

Our study indicates a frequency of IAT experiences in internship courses by medical students about twice as often than by civil engineering students or teaching degree students, with a comparable sensitivity of the students in all three groups. Both form and perceived severity of IAT are similar among the study programs; specific for medical students appears to be frequent working “without adequate safety precautions or safety instructions”.

Comparisons between different study programs are only scarcely reported. Our results compare well to the findings of Rautio et al. [31] in Finland, who compared medical students with students from natural sciences, arts and humanities, technical studies and teaching studies and also found more IAT experiences in medical students as compared to other faculties.

For IAT specific to medicine more studies have been published (see table 1 (Tab. 1)). Even correcting for problems in translation with non-identical meanings for terms indicating the “same” IAT the reported prevalences are in good agreement and describe a problem with IAT in the majority of medical students.

In contrast to Gagyor et al. [5] evaluating the burden by IAT incidences our study differentiated the severity of experienced IAT grading the severity in “not severe”, “rather not severe”, “rather severe” als “severe”. If these parameters, i.e. burden and severity of IAT, are not included in the evaluation prevalences of IAT may vary widely. 

Even if “non severe IAT” are excluded (see table 2 (Tab. 2)) approximately half of all medical students will experience IAT during his/her internships. The high prevalence of IAT in medical students as compared to the other two study programs appears to be independent of the lower limit threshold considered relevant to report an adverse incident as a IAT.

No relevant differences in the type of IAT were seen between the study programs. However, it is remarkable and a cause of concern that 56.7% of medical students report “working without sufficient safety precautions” (40.6%) and/or being required to do activities not permitted for students (40.2%). Unfortunately, we did not specify the type of activities which were requested from medical students.

Regarding “potentially dangerous activities” Sheehan et al. inquired in 1990 [4] whether medical students had been exposed to “unneccesary medical risks“ (personal risks) by other persons (physicians, nurses); Sheehan et al. report an incidence of 44% in good agreement with our findings. A typical example mentioned by Sheehan was collecting blood from a patient with AIDS. Since these or comparable activities are much less likely in both of the study programs we selected for comparison this may explain the differences between medical students and other students concerning “dangerous tasks”; in this case the high pevalence in medical studies would be due to the comparably high likelihood to be tasked with a potentially dangerous activity in medicine, but not by a better management of comparable risks in other study programs or occupations.

The aspect of “potentially dangerous activities” was also studied by Kager et al. in their survey in medical students in 2015 [32]. 49,7% of their participants indicated to have performed tasks during their internship that they have not (yet) been qualified to perform, like drawing blood, placing infusion lines, changing wound dressing, parental drug applications or patient education. Although the advantages of performing these tasks during internships can be rationalized especially in a stressful clinic environment [33], [34] disadvantages even resulting in a potential patient death prevail [35]. The possibility to delegate work to interns who are not qualified or legally permitted to perform these tasks appears to be negligible in civil engineering or teaching studies; to delegate such a task (e.g. grading a pupil or signing a static calculation by an intern) would have a lower benefit for the supervisor concerning time and effort, particularly compared to the risk of (legal) consequences to the supervisor him/herself.

The higher prevalence of “potentially dangerous activities” in medical internships does not completely explain the much higher prevalence of IAT in medical studies which remains elevated even after correction for IAT forms specific in medial studies (c.f. table 2 (Tab. 2)). 

This study is a comprehensive survey in mandatory courses of the three study programs resulting in a high return rate. The good comparability of IAT between the three study programs as well as the corresponding results with the study of Gagyor et al. [5] in medical students at the university of Göttingen with a similar questionnaire underlines the reliabilty of our results. 

Additionally, by collecting data about personality traits and judgement of specific potential IAT situations (results not shown) we could show that the slightly differently distributed peronality traits across the students did not have a relevant influence on the answer patterns and that the judgement of typical potential IAT situations did differ only marginally between the cohorts. Specifically there was no indication for increased sensitivity of medical students toward experiencing and feeling IAT.

It appears to be more problematic to compare data across cultural and linguistic barriers. Different meanings and connotations e.g. among German and English research terms lead to a lack of linguistic precision. We tried to minimize this phenomenon by definitions given for certain terms in order to improve the comparability with international studies.

Interpreting our results also has to consider that the far reaching definition of IAT as used in our study will result in a low threshold for defining an encounter as IAT and will result in a high incidence rate. This also will add a variety of different incidents and of incidents with different gravity as equal in the sum of IATs. Judging the IAT severity enables a grading of the feeling of an encounter as IAT but will solely be a subjective feeling due to the questionnaire type of these studies.

Lastly the internships of each study program have an own characteristic environment that has to be factored in an overarching comparison. Medical students will experience contacts with a multitude of people (physicians, nurses, etc.) in a hospital setting resulting in a high possibility of an IAT encounter among them. During an internship in an engineering office or at a school where the number of contacts per student will usually be smaller, the risk of an IAT encounter will be less likely. Also the lack of personnel in German hospitals has been proven to create a high level of frustration and stress in its employees as compared to other occupations [27], additionally increasing the IAT risk. In contrast, the topic of “potentially dangerous activities” which is nearly specific for medical internships appears to be a less important factor for the incidence of IAT.

## 5. Conclusion

IAT in study internships appears to be a much larger problem in medicine as compared to other professions. Medical students report IAT in internships twice as often as teaching degree students or civil engineering students. Demeaning comments or lack of communication by hierarchical superiors is given as a major factor. Additionally, most students (>50%) report to be tasked in patient care outside of their competencies or with insufficient instructions – endangering both patients and students. 

Therefore it is necessary to improve teaching conditions in medical studies by remediating the causes of IAT (see introduction). In order to achieve this different authors have suggested multiple levels of action: the practice competency of medical students has to be improved at early stages [12], [36], e.g. by information on IAT or possible actions if IAT is encountered [37], discussions with peers or role encounters to prepare for and cope with IAT [38]. Secondly, university staff and hospital instructors should be sensibilized for potential IAT encounters and trained in respectful, professional and appreciative student contacts [9], [12], [27], since as superiors they have a major influence on a productive learning environment [39] and report rates of IAT incidences [40]. Thirdly, organisational improvements are necessary [30], both by a faculty and a teaching hospital, e.g. the goodwill to counteract IAT, time slots reserved for teaching [7] and a separate salary specifically labelled for teaching duties, organizing information and training sessions, supervising the prevalence of IAT by evaluation and introducing a registry for anonymously reporting IAT with the option to pursue these incidences if necessary [37], [41], [42]. 

Most importantly, in our experience organizational changes are considered necessary; only changes in the professional culture, a flat hierarchy and support of a productive teaching environment by faculty and teaching hospitals will result in a lasting decrease of IAT. Fried et al. [37] and Lind et al. [41] have reported interventional studies over 13 and 6 years resp. with extensive interventions in all three levels. They report a decrease of IAT at their faculties by ~25%, from a total of 75% to 57% [31] and by ~33% from 62,9% to 40,3% [41]. This indicates a slow change of professional attitude but underlines the possibility of successful interventions in the long run.

## Acknowledgement

We thank Prof. Dr. Wolfgang Himmel, Institute of Family Medicine, Medical Faculty, University Göttingen for permission to use their questionnaire; we thank Prof. Dr. Rolf Katzenbach, Ms. Solenne Rochée and Mr. Sebastian Fischer, Institute of Geotechnique, Technical University Darmstadt, and Mr. Alexander Storch and all colleagues at the Institute of High School Pedagogy, Goethe-University Frankfurt, for their support in data collection in their respective study programs. We also appreciate many helpful discussions by Dr. Keno Krewer. 

## Abbreviations

- civ eng: civil engineering, student of civil engineering
- TD: teaching degree, student of (high school) teaching degree
- Med: medicine, medical student
- IAT: inadequate treatment, mistreatment
- IAT yes: having experience with inadequate treatment
- IAT no: without experience with inadequate treatment

## Competing interests

The authors declare that they have no competing interests. 

## Supplementary Material

Questionnaire in german

Tables S2, S3, S5-S9 and figure S4

Figure S4 and table S9

## Figures and Tables

**Table 1:**
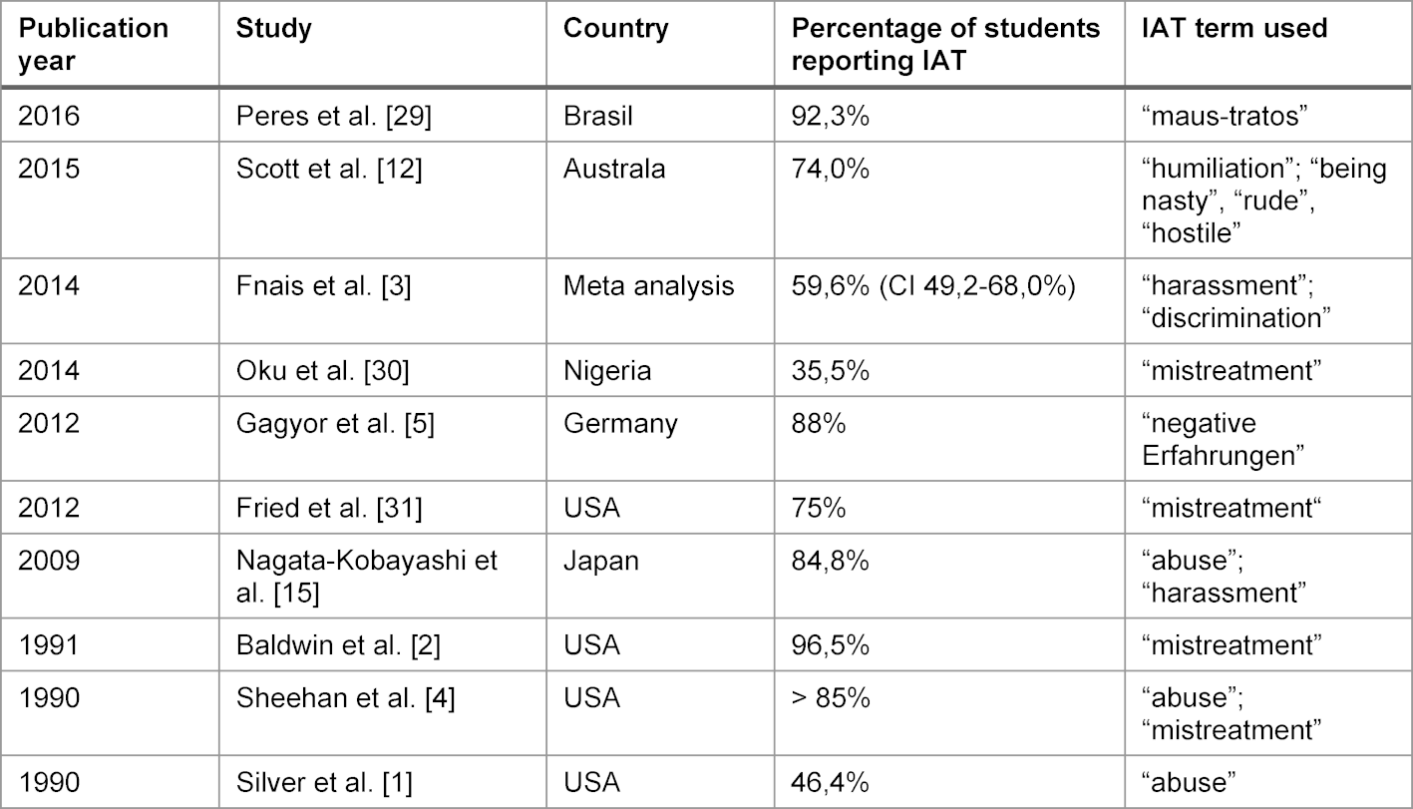
Comparison of IAT frequency in medical education in different studies with special consideration of the IAT terms used

**Table 2:**
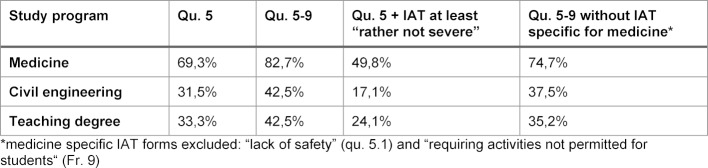
Comparison of percentages of students with personal IAT experiences using different threshold criteria for IAT grading
