# Supplementary material for: Inadequate treatment in internships: a comparison between medical and other students
Source: GMS J Med Educ. 2021 Feb 15;38(2):Doc45. doi: 10.3205/zma001441 (PMC7958909; doi:10.3205/zma001441)
Supplement: Questionnaire in german [file JME-38-2-45-s-001.pdf]

Unangemessene Behandlung von Studenten in der praktischen Ausbildung: Ein Vergleich zwischen verschiedenen Berufen

Das Institut für Arbeits-, Umwelt- und Sozialmedizin am Fachbereich Medizin der Goethe-Universität Frankfurt am Main führt eine Studie durch, in der die Wahrnehmung von unangemessenem Verhalten in der praktischen Ausbildung zwischen verschiedenen Studiengängen und Persönlichkeitstypen verglichen werden soll.

Als Erhebungsinstrument dient dieser Fragebogen. Die damit gewonnenen Daten sind anonym und werden ausschließlich im Rahmen der Studie verwendet.

Die Teilnahme an der Studie ist freiwillig. Wenn Sie nicht teilnehmen möchten, geben Sie den Fragebogen einfach unausgefüllt wieder ab.

Jeder einzelne ausgefüllte Fragebogen ist uns jedoch eine große Hilfe und trägt dazu bei, uns ein besseres Bild über die Situation in den jeweiligen Studiengängen zu vermitteln. Deshalb würden wir uns sehr über Ihre Teilnahme freuen.

Hinweise:

Folgende Antwortformate werden in diesem Fragebogen verwendet:

1. Single Choice: Auswahl nur einer Option	☒ Ja ❍ Nein ❍ Vielleicht
2. Multiple Choice: Auswahl mehrerer Optionen möglich	❑ Vorspeise ☒ Hauptgang ☒ Nachtisch
3. Freitextfeld: Beantwortung der Frage in eigener Formulierung	Man sollte mehr Käse als Dessert anbieten
4. Antwortbalken: Einschätzung innerhalb der je gegebenen Extrembereiche durch einen senkrecht gezogenen Strich	schlecht ——|—— gut

Zugunsten der besseren Lesbarkeit wird in diesem Fragebogen auf die Spezifizierung des Geschlechts verzichtet und das generische Maskulinum (z.B. Student statt StudentIn) verwendet, wenn das Geschlecht der Person für die Beantwortung der Frage nicht von Relevanz ist.

Ansprechpartner:

Sonia Bormuth<br>Studienkoordinatorin<br>s6177353@stud.uni-frankfurt.de	Prof. Dr. Johannes Schulze<br>Projektleiter<br>j.schulze@em.uni-frankfurt.de

ASU – Institut für Arbeits-, Umwelt- und Sozialmedizin der Goethe-Universität Frankfurt am Main

Attachment 11 to: Bormuth S, Ackermann H, Schulze J. *Inadequate treatment in interships: a comparison between medical and other students.* GMS J Med Educ. 2021;38(2):Doc45. DOI: 10.3205/001441

TEIL A – Unangemessene Behandlung

„Unangemessene Behandlung“ bezeichnet in diesem Fragebogen ein Verhalten, das in seiner Art oder seinem Ausmaß von mindestens einer der in der Situation anwesenden Personen als nicht angebracht oder unpassend empfunden wird und infolgedessen Gefühle von Überraschung, Ärger, Scham oder Enttäuschung auslöst.

Ob ein Verhalten wie z.B. eine harsche Zurechtweisung angemessen oder unangemessen ist, hängt damit von der Situation ab, in der es auftritt, sowie von der Bewertung der Handlung durch die beteiligten Personen.

Dieser Fragebogen bezieht sich ausschließlich auf die unangemessene Behandlung **durch andere im Krankenhaus oder der Praxis beschäftigte Personen**, nicht auf solche durch z.B. Patienten.

1. Wie zufrieden sind Sie mit Ihrem Studienfach?

   sehr unzufrieden ——————|—————— sehr zufrieden

2. Wie zufrieden sind Sie mit den Praktika (z.B. Pflegepraktika, Blockpraktika, Famulaturen) in Ihrem Studienfach?

   sehr unzufrieden ——————|—————— sehr zufrieden

3. Wie würden Sie die Lernatmosphäre in Ihrer praktischen Ausbildung anhand Ihrer bisherigen Erfahrungen beschreiben?

   sehr unzufrieden ——————|—————— sehr zufrieden

4. Als wie häufig würden Sie das Vorkommen von unangemessener Behandlung in der praktischen Ausbildung Ihres angestrebten Berufes im Vergleich zu der in anderen Berufen einschätzen?

   sehr viel seltener ——————|—————— sehr viel häufiger

Attachment 11 to: Bormuth S, Ackermann H, Schulze J. *Inadequate treatment in interships: a comparison between medical and other students.* GMS J Med Educ. 2021;38(2):Doc45. DOI: 10.3205/001441

5. Sind Sie während Ihrer **Praktika** schon einmal unangemessen behandelt worden (z.B. durch Aufgaben ohne Lerneffekt, Bloßstellung oder Nichtbeachtung)?

❍ Ja ❍ Nein

Wenn nein, bitte überspringen Sie die folgenden Unterfragen und fahren Sie mit Frage 6 fort.

5.1. **In welcher Form** fand die unangemessene Behandlung statt? *[Mehrfachantworten möglich]*

5.2. Wer war **am häufigsten** der Verursacher dieser Form der unangemessenen Behandlung? *[nur eine Nennung pro Zeile]*

↓			Arzt	anderes medizinisches Personal	anderer Student	Sonstige
		**VERBAL**				
❑	1	Anschreien / rüdes Zurechtweisen	❍	❍	❍	❍
❑	2	negative oder beleidigende Aussage über Berufliches (z.B. Karrierechancen, Kompetenz)	❍	❍	❍	❍
❑	3	negative oder beleidigende Aussage über Privates (z.B. Aussehen oder Verhalten)	❍	❍	❍	❍
❑	4	Vorwurf, andere bei der Arbeit zu stören	❍	❍	❍	❍
❑	5	Bloßstellung oder Demütigung vor anderen	❍	❍	❍	❍
		**NONVERBAL**				
❑	6	Nichtbeachtung / Ignorieren	❍	❍	❍	❍
❑	7	abwertendes oder beleidigendes Verhalten (z.B. Gestik, Mimik, Hinterherpfeifen)	❍	❍	❍	❍
❑	8	Nichtwahrung körperlicher Distanz (z.B. den Arm tätscheln, auf den Po klopfen, ins Ohr flüstern)	❍	❍	❍	❍
		**ORGANISATORISCH**				
❑	9	Aufgaben ohne Lerneffekt	❍	❍	❍	❍
❑	10	schlechte Betreuung	❍	❍	❍	❍
❑	11	Arbeit ohne ausreichende Sicherheitsvorkehrungen bzw. Einweisung	❍	❍	❍	❍
❑	12	schlechte Noten oder Bewertungen	❍	❍	❍	❍
❑	13	Überforderung durch Aufgaben	❍	❍	❍	❍
❑	14	Ausgeben meiner Leistung als die eines anderen	❍	❍	❍	❍

Attachment 11 to: Bormuth S, Ackermann H, Schulze J. *Inadequate treatment in interships: a comparison between medical and other students.* GMS J Med Educ. 2021;38(2):Doc45. DOI: 10.3205/001441

❑ 15 **SONSTIGES**: ____________________________ ❍ ❍ ❍ ❍

5.3. Wie häufig sind Sie in den **letzten 12 Monaten** während Ihrer praktischen Ausbildung unangemessen behandelt worden?

❍ kein Mal ❍ 1 Mal ❍ 2-3 Male ❍ mindestens 4 Male

❍ Ich hatte keine Praktika in diesem Zeitraum.

5.4 In welchem Fachgebiet fand die unangemessene Behandlung **am häufigsten** statt?

❍ Chirurgie ❍ Gynäkologie ❍ HNO / Augenheilkunde / Urologie

❍ Innere Medizin ❍ Neurologie ❍ Psychiatrie

❍ Sonstige: _____________

5.4. Zu welchen der unter 5.1. genannten Formen zählt Ihre **gravierendste** Erfahrung unangemessener Behandlung?

❍ 1 ❍ 2 ❍ 3 ❍ 4 ❍ 5 ❍ 6 ❍ 7 ❍ 8

❍ 9 ❍ 10 ❍ 11 ❍ 12 ❍ 13 ❍ 14 ❍ 15

5.5. Wie schwerwiegend war die psychische Auswirkung der für Sie **gravierendsten** unangemessenen Behandlung?

❍ gar nicht schwerwiegend	(Vorfall direkt danach innerlich abgehakt)
❍ eher nicht schwerwiegend	(einige Tage darüber nachgedacht oder in schlechterer Stimmung als üblich gewesen, danach innerlich abgehakt)
❍ eher schwerwiegend	(innerlich noch nicht abgehakt, emotionale Erinnerungen mit Einfluss auf Verhalten (Rückzug, Vermeidung ähnlicher Situationen oder Kontakt mit Verursacher))
❍ sehr schwerwiegend	(psychotherapeutische Hilfe in Anspruch genommen)

5.6. Wie haben Sie darüber hinaus auf die für Sie **gravierendste** unangemessene Behandlung reagiert? *[Mehrfachantworten möglich]*

❑ Krankschreibung oder Wechsel der Praktikumsstelle	❑ Ansprechen des Vorfalls gegenüber dem Verursacher	❑ Ansprechen des Vorfalls gegenüber einer zuständigen Meldestelle oder Ansprechperson	❍ nichts davon

Attachment 11 to: Bormuth S, Ackermann H, Schulze J. *Inadequate treatment in interships: a comparison between medical and other students.* GMS J Med Educ. 2021;38(2):Doc45. DOI: 10.3205/001441

6. Sind Sie während Ihrer praktischen Ausbildung schon einmal von einem **Vorgesetzten aus sexistischer Motivation** (also aufgrund Ihres Geschlechts) heraus unangemessen behandelt worden?

❍ Ja, und zwar: ❑ verbal ❑ nonverbal ❑ organisatorisch ❑ Sonstiges: __________

❍ Nein

7. Sind Sie während Ihrer praktischen Ausbildung schon einmal von einem **Vorgesetzten aus rassistischer Motivation** heraus unangemessen behandelt worden?

❍ Ja, und zwar: ❑ verbal ❑ nonverbal ❑ organisatorisch ❑ Sonstiges: __________

❍ Nein

8. Sind Sie während Ihrer praktischen Ausbildung schon einmal von einem **Vorgesetzten aufgrund Ihrer angestrebten Spezialisierungsrichtung** unangemessen behandelt worden?

❍ Ja, und zwar: ❑ verbal ❑ nonverbal ❑ organisatorisch ❑ Sonstiges: __________

❍ Nein

9. Sind Sie während Ihrer praktischen Ausbildung schon einmal von einem **Vorgesetzten** zur Ausführung einer (bei Ihrem damaligen Ausbildungsstand) **nicht erlaubten Tätigkeit** (z.B. Kreuzblutentnahme für EK.s, Erstgabe von Antibiotika, Aufklärungsgespräch bei OP) aufgefordert worden?

❍ Ja ❍ Nein

10. Was verstärkt Ihrer Meinung nach das Vorkommen von unangemessener Behandlung in der praktischen Ausbildung? *[Beantwortung optional]*

11. Was wirkt Ihrer Meinung nach dem Vorkommen von unangemessener Behandlung in der praktischen Ausbildung entgegen? *[Beantwortung optional]*

12. Von universitärer Seite aus sollte über den Umgang mit oder die Handlungsmöglichkeiten bei unangemessener Behandlung in der praktischen Ausbildung informiert werden, z.B. im Rahmen einer allgemeinen schriftlichen Mitteilung oder einer allgemeinen Lehrveranstaltung.

stimme gar nicht zu ——————|—————— stimme vollkommen zu

Attachment 11 to: Bormuth S, Ackermann H, Schulze J. *Inadequate treatment in interships: a comparison between medical and other students.* GMS J Med Educ. 2021;38(2):Doc45. DOI: 10.3205/001441

Im Folgenden werden kurz beispielhafte spezifische Situationen geschildert, wie sie während des Praktikums auftreten können. Bitte bewerten Sie bei diesen Beispielen das Verhalten des Vorgesetzten:

13. Ein Student kommt wiederholt zu spät zur wöchentlichen Besprechung. Er wurde bisher nicht von seinem Vorgesetzten angehalten, pünktlich zu sein, wird von diesem aber nun vor den anderen Anwesenden zurechtgewiesen.

gar nicht angemessen ——— | ——— vollkommen angemessen

14. Ein Student ist mit einer Aufgabe betraut worden. Ein betreuender Vorgesetzter kommt vorbei und sieht, dass er sie viel komplizierter bearbeitet als nötig wäre. Weil er den Studenten aber nicht vor den anderen im Raum anwesenden Studenten korrigieren will, sagt er nichts.

gar nicht angemessen ——— | ——— vollkommen angemessen

15. Ein Vorgesetzter beauftragt einen Studenten damit, mit der Teamkasse zum Supermarkt zu gehen und für alle Mittagessen zu holen.

gar nicht angemessen ——— | ——— vollkommen angemessen

16. Eine kopftuchtragende Studentin setzt bei ihrem Vorgesetzten durch, dass sie während ihres Praktikums einige Aufgaben übertragen bekommt, um die sie sich komplett eigenverantwortlich kümmern darf. Nach dem Gespräch meint der Vorgesetzte abschließend anerkennend: „Sie haben ja eine Durchsetzungskraft. Das hätte ich Ihnen mit Ihrem Kopftuch gar nicht zugetraut."

gar nicht angemessen ——— | ——— vollkommen angemessen

17. Ein Student kommt wiederholt zu spät zur wöchentlichen Besprechung. Er wurde bereits mehrmals von seinem Vorgesetzten dazu angehalten, pünktlich zu sein, und wird nun von diesem vor den anderen Anwesenden zurechtgewiesen.

gar nicht angemessen ——— | ——— vollkommen angemessen

18. Nach dem Unterzeichnen des Dokuments über die pflichtgemäße Ableistung des Praktikums fragt die Vorgesetzte, ob der männliche Student Lust habe, sich am Abend mit ihr auf einen Drink zu treffen, nun, wo er sein Praktikum hier beendet habe.

gar nicht angemessen ——— | ——— vollkommen angemessen

19. Für Renovierungsarbeiten müssen die Bürozimmer ausgeräumt werden, Schreibtische, Stühle, Computer, Aktenschränke, Aktenordner und Rollkommoden. Als sich das Team am Morgen zur Organisation der Umräumarbeiten trifft, erklärt der Vorgesetzte, dass die weiblichen Angestellten nicht helfen und in der Kaffeküche Pause machen sollten. Die Männer würden das Tragen der Einrichtungsgegenstände alleine erledigen.

gar nicht angemessen ——— | ——— vollkommen angemessen

Attachment 11 to: Bormuth S, Ackermann H, Schulze J. *Inadequate treatment in interships: a comparison between medical and other students.* GMS J Med Educ. 2021;38(2):Doc45. DOI: 10.3205/001441

Bitte bewerten Sie jetzt das Verhalten des Studenten:

20. Bei einer Tätigkeit unterläuft dem Vorgesetzten ein Fehler. Der ihn begleitende Student, der alles mitverfolgt, macht ihn in Gesprächslautstärke darauf aufmerksam. Es sind noch zwei Kollegen des Vorgesetzten im Raum, die das Gespräch mithören.

gar nicht angemessen ——————|—————— vollkommen angemessen

21. Man ist gerade emsig am Arbeiten. Eine fremde Person, offenbar einer der neuen Studenten, tritt ein und stellt sich mit lauter Stimme vor.

gar nicht angemessen ——————|—————— vollkommen angemessen

22. Der Vorgesetzte gibt dem neuen Studenten eine Aufgabe, die dieser aufgrund seines Ausbildungsstandes schon bearbeiten können sollte. Der Student, der sich nicht zutraut, die Aufgabe zu erledigen, bittet den Vorgesetzten, die Aufgabe mit ihm gemeinsam auszuführen, bis er sich in der eigenständigen Bearbeitung sicher genug fühle.

gar nicht angemessen ——————|—————— vollkommen angemessen

23. Ein Student hat eine Aufgabe bekommen. Als der Vorgesetzte vorbeikommt, bemerkt er, dass der Student bei der Bearbeitung der Aufgabe etwas übersehen hat. Er macht den Studenten in sachlichem Ton auf den Fehler aufmerksam und holt die anderen im Raum befindlichen Studenten für seine Erklärung hinzu, damit auch sie lernen, warum diese Aufgabe auf die eine, aber nicht die andere Weise erledigt werden kann. Im Anschluss äußert der Student unter vier Augen gegenüber dem Vorgesetzten, dass er sich durch dessen Verhalten vorgeführt und gedemütigt gefühlt habe und bittet ihn, ihn in Zukunft nicht mehr vor anderen zu korrigieren.

gar nicht angemessen ——————|—————— vollkommen angemessen

24. Ein Student zeigt im Vergleich zu den anderen weniger Motivation. Deshalb nimmt der Vorgesetzte stets seine motivierteren Kommilitonen zu interessanten Gesprächen mit. Daraufhin beschwert sich der Student, gegenüber den anderen Studenten benachteiligt zu werden.

gar nicht angemessen ——————|—————— vollkommen angemessen

25. Man ist gerade emsig am Arbeiten. Eine fremde Person, offenbar einer der neuen Studenten, tritt ein und verharrt schweigend und abwartend mehrere Minuten in der Nähe der Tür.

gar nicht angemessen ——————|—————— vollkommen angemessen

26. Ein Student hat seine Aufgabe unwissentlich falsch ausgeführt und seinem ohnehin gestressten Vorgesetzten damit zusätzliche Arbeit bereitet. Der Vorgesetzte wird daraufhin wütend und er schreit den Studenten an: „Einen so inkompetenten Praktikanten habe ich meinen Lebtag noch nicht gehabt!

Sie sind wirklich eine Schande für alle Medizinstudenten!" Der Student meldet den Vorfall der zuständigen Stelle und bittet um einen anderen Praktikumsplatz.

gar nicht angemessen ——————— vollkommen angemessen

TEIL B – Hierarchie

„Hierarchie" beschreibt in unserem Fragebogen die Bildung einer Abstufung oder Rangfolge zwischen verschiedenen Personen am Arbeits- bzw. Ausbildungsplatz. Diese Abstufung oder Rangfolge wirkt sich auf die Interaktion zwischen Personen verschiedener Hierarchiestufen aus.

Mit „flacher Hierarchie" ist in diesem Fragebogen ein geringer Abstand zwischen zwei aufeinanderfolgenden hierarchischen Stufen gemeint, mit „steiler Hierarchie" ein großer Abstand zwischen zwei aufeinanderfolgenden hierarchischen Stufen.

27. Wie schätzen Sie im Großen und Ganzen die Hierarchie in Ihrem angestrebten Beruf anhand Ihrer bisherigen praktischen Erfahrungen ein? Als

sehr flach ——————— sehr steil

28. Welches Hierarchieniveau halten Sie in Ihrem Beruf für optimal?

sehr flach ——————— sehr steil

29. Wie schätzen Sie im Großen und Ganzen die Hierarchie in den folgenden Berufen ein?

Bauingenieur-wesen: sehr flach ——————— sehr steil

Lehramt: sehr flach ——————— sehr steil

Polizei: sehr flach ——————— sehr steil

30. Welches Hierarchieniveau halten Sie in den folgenden Berufen für optimal?

Bauingenieur-wesen: sehr flach ——————— sehr steil

Lehramt: sehr flach ——————— sehr steil

Polizei: sehr flach ——————— sehr steil

31. Inwiefern wirkt sich in Ihren Augen die vorherrschende Hierarchie auf das Vorkommen von unangemessener Behandlung aus? Eine steile Hierarchie

Attachment 11 to: Bormuth S, Ackermann H, Schulze J. *Inadequate treatment in interships: a comparison between medical and other students.* GMS J Med Educ. 2021;38(2):Doc45. DOI: 10.3205/001441

schützt vor unangemessener Behandlung		fördert unangemessene Behandlung

Bitte schätzen Sie Ihren Beruf anhand Ihrer bisherigen Erfahrungen in Bezug die folgenden Kriterien ein:

32. Der Umgangston ist in der Regel:

freundschaftlich-locker		formell-streng

33. Bezüglich betrieblicher Regeln gilt:

Es gelten die gleichen Regeln für alle Mitarbeiter.		Vorgesetzte müssen sich weniger an die Regeln halten als ihre Mitarbeiter.

34. Zuständigkeiten (Wer kümmert sich um welche anfallende Arbeit?) sind meist:

unklar oder auszuhandeln		klar geregelt

35. Verantwortlichkeiten (Wer übernimmt die Verantwortung für die geleistete Arbeit?) sind meist:

unklar oder auszuhandeln		klar geregelt

36. Bei der Bearbeitung der einem zugewiesenen Aufgaben

hat man in der Regel freie Hand.		ist man meist auf Begutachtung oder Abzeichnung durch den Vorgesetzten angewiesen.

37. Der Machtunterschied zwischen zwei aufeinanderfolgenden hierarchischen Ebenen ist:

sehr klein		sehr groß

38. Das Verhalten im Umgang mit einem Vorgesetzten ist

gleich respektvoll wie mit einem Kollegen.		sehr viel respektvoller als mit einem Kollegen.

39. Das Überdenken und ggf. Hinterfragen von Anweisungen vor Ausführung ist:

sehr erwünscht		gar nicht erwünscht

40. Wenn Mitarbeiter bemerken würden, dass ihrem Vorgesetzten ein Fehler unterlaufen ist, würden sie sich

in jedem Fall trauen, ihn darauf anzusprechen.	——————+——————	in keinem Fall trauen, ihn darauf anzusprechen.

41. Die Gruppe betreffende Entscheidungen werden in der Regel getroffen:

❍ demokratisch durch die Gruppe ❍ durch den Vorgesetzten unter Einbezug der Gruppe ❍ durch den Vorgesetzten allein

TEIL C – Persönlichkeitsmerkmale

**Inwieweit treffen die folgenden Aussagen auf Sie zu?**	überhaupt nicht zutreffend	eher nicht zutreffend	weder noch	eher zutreffend	sehr zutreffend
42. Ich bin eher zurückhaltend, reserviert.	❍	❍	❍	❍	❍
43. Ich neige dazu, andere zu kritisieren.	❍	❍	❍	❍	❍
44. Ich erledige Aufgaben gründlich.	❍	❍	❍	❍	❍
45. Ich schenke anderen leicht Vertrauen, glaube an das Gute im Menschen.	❍	❍	❍	❍	❍
46. Ich bin bequem, neige zur Faulheit.	❍	❍	❍	❍	❍
47. Ich bin entspannt, lasse mich durch Stress nicht aus der Ruhe bringen.	❍	❍	❍	❍	❍
48. Ich habe eine aktive Vorstellungskraft, bin phantasievoll.	❍	❍	❍	❍	❍
49. Ich gehe aus mir heraus, bin gesellig.	❍	❍	❍	❍	❍
50. Ich werde leicht nervös und unsicher.	❍	❍	❍	❍	❍
51. Ich habe nur wenig künstlerisches Interesse.	❍	❍	❍	❍	❍

Bitte kreuzen Sie an, in welchem Maße Sie mit den angegebenen Aussagen übereinstimmen. Das Ausmaß der Übereinstimmung wird hier angegeben nach:

1= trifft (für mich) überhaupt nicht zu
5 = unentschieden
9 = trifft (für mich) vollkommen zu

	**1**	**2**	**3**	**4**	**5**	**6**	**7**	**8**	**9**
52. Ich neige dazu, andere zu manipulieren, um meinen Willen durchzusetzen.	❍	❍	❍	❍	❍	❍	❍	❍	❍
53. Ich neige dazu, keine Gewissensbisse zu haben.	❍	❍	❍	❍	❍	❍	❍	❍	❍
54. Ich neige dazu, von anderen bewundert zu werden.	❍	❍	❍	❍	❍	❍	❍	❍	❍

Attachment 11 to: Bormuth S, Ackermann H, Schulze J. *Inadequate treatment in interships: a comparison between medical and other students.* GMS J Med Educ. 2021;38(2):Doc45. DOI: 10.3205/001441

55. Ich habe getäuscht oder gelogen, um meinen Willen durchzusetzen.	❍ ❍ ❍ ❍ ❍ ❍ ❍ ❍ ❍
56. Ich neige dazu, mich nicht um die Moral meiner Handlungen zu kümmern.	❍ ❍ ❍ ❍ ❍ ❍ ❍ ❍ ❍
57. Ich neige dazu, von anderen Menschen beachtet werden zu wollen.	❍ ❍ ❍ ❍ ❍ ❍ ❍ ❍ ❍
58. Ich habe Schmeicheleien genutzt, um meinen Willen durchzusetzen.	❍ ❍ ❍ ❍ ❍ ❍ ❍ ❍ ❍
59. Ich neige dazu, gefühllos und unsensibel zu sein.	❍ ❍ ❍ ❍ ❍ ❍ ❍ ❍ ❍
60. Ich neige dazu, nach Ansehen oder Status zu streben.	❍ ❍ ❍ ❍ ❍ ❍ ❍ ❍ ❍

TEIL D – Demografische Daten

61. Welches Geschlecht haben Sie?

❍ weiblich ❍ männlich ❍ anders

62. Wie alt sind Sie?

❍ bis einschließlich 20 Jahre ❍ 21 bis 25 Jahre ❍ 26-30 Jahre

❍ 31 bis 35 Jahre ❍ älter als 35 Jahre

63. In welchem Fachgebiet wollen Sie später arbeiten?

❍ Chirurgie ❍ Gynäkologie ❍ HNO / Augenheilkunde / Urologie

❍ Innere Medizin ❍ Neurologie ❍ Psychiatrie

❍ unentschlossen ❍ Sonstiges: ___________

64. Was ist Ihre Nationalität?

65. Was ist Ihre Muttersprache?

66. Was ist die Muttersprache Ihrer Eltern?

Vater:	Mutter:

67. In welchem Land haben Sie bisher die längste Zeit Ihres Lebens gelebt?

68. Wie streng sind Sie erzogen worden?

gar nicht streng ——————|—————— sehr streng

Attachment 11 to: Bormuth S, Ackermann H, Schulze J. *Inadequate treatment in interships: a comparison between medical and other students.* GMS J Med Educ. 2021;38(2):Doc45. DOI: 10.3205/001441

69. Wenn Sie Anmerkungen oder Kommentare zu diesem Fragebogen haben, bitte teilen Sie sie hier mit:

Hiermit endet der Fragebogen. Wir danken Ihnen sehr herzlich für Ihre Teilnahme!

Attachment 11 to: Bormuth S, Ackermann H, Schulze J. *Inadequate treatment in interships: a comparison between medical and other students.* GMS J Med Educ. 2021;38(2):Doc45. DOI: 10.3205/001441
