# Supplementary material for: Inadequate treatment in internships: a comparison between medical and other students
Source: GMS J Med Educ. 2021 Feb 15;38(2):Doc45. doi: 10.3205/zma001441 (PMC7958909; doi:10.3205/zma001441)
Supplement: Tables S2, S3, S5-S9 and figure S4 [file JME-38-2-45-s-002.pdf]

Table S2: Comparison of demographic characteristica in participants from three study programs

	Medicine N (%)	Civil engineering N (%)	Teaching degree N (%)	Total N (%)
**Sex**				
- female	163 (62%)	54 (37%)	62 (71%)	279 (57%)
- male	90 (35%)	85 (58%)	24 (28%)	199 (40%)
- other	0 (0%)	2 (1%)	0 (0%)	2 (0%)
- no data	8 (3%)	5 (3%)	1 (1%)	14 (3%)
**Age:**				
- ≤ 20 years	65 (25%)	25 (17%)	33 (38%)	123 (25%)
- 21-25 years	151 (58%)	106 (73%)	35 (40%)	292 (59%)
- 26-30 years	25 (10%)	10 (7%)	10 (12%)	45 (9%)
- 31-35 years	10 (4%)	0 (0%)	4 (5%)	14 (3%)
- > 35 years	2 (1%)	0 (0%)	4 (5%)	6 (1%)
- no data	8 (3%)	5 (3%)	1 (1%)	14 (3%)
**Cultural background:**				
- German	203 (78%)	119 (82%)	72 (83%)	394 (90%)
- non-German	47 (18%)	20 (14%)	13 (15%)	80 (16%)
- no data	11 (4%)	7 (5%)	2 (2%)	20 (4%)

All percentage values are based on all participants = 100%

Table S3: IAT frequencies among students from three study programs.

	Civil engineering N (%)	Medicine N (%)	Teaching degree N (%)
**Question 5 = yes**	46 (32%)	181 (69%)	29 (33%)
**Questions 6 – 9 = yes**	16 (11%)	35 (13%)	8 (9%)
**no**	84 (58%)	45 (17%)	50 (57%)

All percentage values are based on all participants = 100%. Incidences are given in absolute and relative values. Question 5 = yes ≙ IAT experience reported in question 5; question 6 – 9 = yes ≙ IAT experience reported only in questions 6-9; no ≙ no IAT reported in questions 5 to 9.

Attachment 2 to: Bormuth S, Ackermann H, Schulze J. *Inadequate treatment in interships: a comparison between medical and other students.* GMS J Med Educ. 2021;38(2):Doc45. DOI: 10.3205/001441

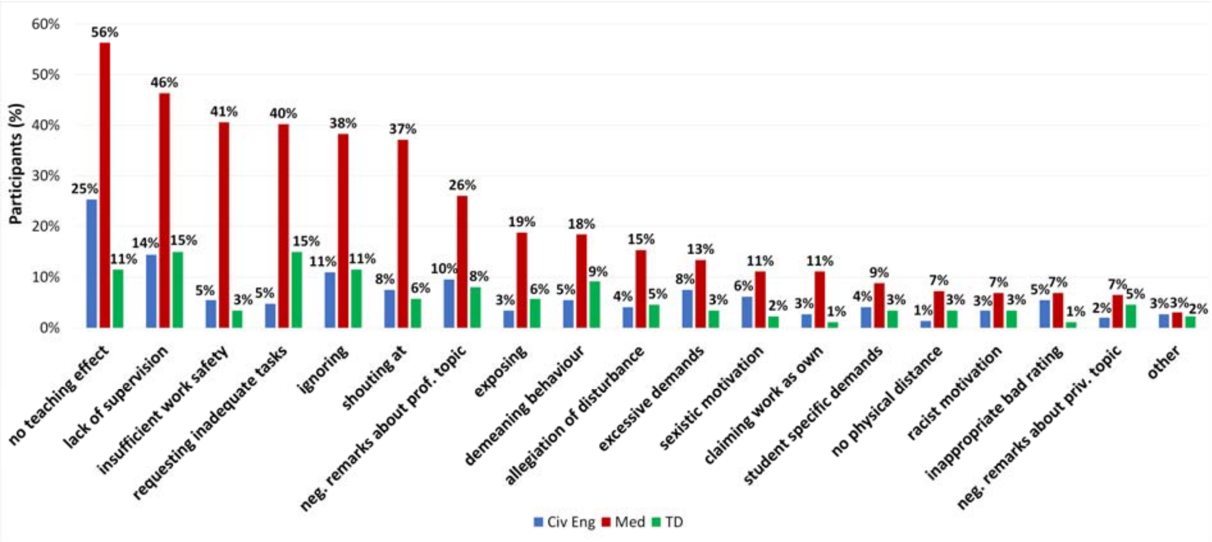

All percentage values are based on all participants = 100%.

Figure S4: Frequency of IAT experience in students from three study programs

Table S5: IAT category frequencies for three study programs.

	Civil engineering N (%)	Medicine N (%)	Teaching degree N (%)
**verbal IAT**	37 (25%)	176 (67%)	28 (32%)
**nonverbal IAT**	23 (16%)	128 (49%)	17 (20%)
**organisational IAT**	45 (31%)	177 (68%)	24 (28%)

Incidences are given in absolute and relative values. Question 9 answers were added to verbal IAT, positive answers in questions 6-8 to the category given by the students as appropriate for their resp. incident.
All percentage values are based on all participants = 100%. Since multiple answers were possible, and some participants did not answer any question the values do not add up to 100%.

Table S6: Forms of IAT sorted by frequency in the study programs.

	Civil engineering N (%)	Medicine N (%)	Teaching degree N (%)
**No teaching effect**	37 (25%)	147 (56%)	10 (12%)
**Lack of supervision**	21 (14%)	121 (46%)	13 (15%)
**Insufficient work safety**	8 (6%)	106 (41%)	3 (3%)
**Requesting inadequate tasks**	7 (5%)	105 (40%)	13 (15%)
**Ignoring**	16 (11%)	100 (38%)	10 (12%)
**Shouting at**	11 (8%)	97 (37%)	5 (6%)
**Negative remarks about professional topic**	14 (10%)	68 (26%)	7 (8%)
**Exposing**	5 (3%)	49 (19%)	5 (6%)
**Demeaning behaviour**	8 (6%)	48 (18%)	8 (9%)
**Allegation of disturbance**	6 (4%)	40 (15%)	4 (5%)
**Excessive demands**	11 (8%)	35 (13%)	3 (3%)
**Claiming work as own**	4 (6%)	29 (11%)	1 (2%)
**Sexistic motivation**	9 (3%)	29 (11%)	2 (1%)
**Student specific demands**	6 (4%)	23 (9%)	3 (3%)
**No physical distance**	2 (1%)	19 (7%)	3 (3%)
**Inappropriate bad rating**	8 (3%)	18 (7%)	1 (3%)
**Racist motivation**	5 (6%)	18 (7%)	3 (1%)
**Negative comment about private topic**	3 (2%)	17 (7%)	4 (5%)
**Other**	4 (3%)	8 (3%)	2 (2%)

Incidences are given in absolute and relative values. All percentage values are based on all participants = 100%. Since multiple answers were possible, and some participants did not answer any question the values do not add up to 100%.

Table S7: P-values and effect sizes in frequency differences for the six most common IAT forms in the study programs civil engineering, medicine and teaching degree

Type of IAT	p-value Fisher-Freeman-Halton test	Effect size ω
No teaching effect	p < 0,0001	0,38
Lack of supervision	p < 0,0001	0,34
Requesting inadequate tasks	p < 0,0001	0,38
Insufficient work safety	p < 0,0001	0,42
Ignoring	p < 0,0001	0,31
Shouting at	p < 0,0001	0,36

Table S8: Different severity levels of the most severe IAT from all IAT experienced

	Civil engineering N (%)	Medicine N (%)	Teaching degree N (%)
**Not severe**	20 (44%)	49 (27%)	8 (28%)
**Rather not severe**	19 (42%)	106 (59%)	12 (41%)
**Rather severe**	6 (13%)	24 (13%)	9 (31%)
**Severe**	0 (0%)	0 (0%)	0 (0%)

All percentage values are based on all participants = 100%.

Table S9: subgroup analysis based on participant sex.

	**Medicine**		**Civil engineering**		**Teaching degree**	
	**F**	**M**	**F**	**M**	**F**	**M**
**IATyes, Qu. 5:**<br>- N_{IATyes}/N_{total}<br>- %	$*_{MedM}$<br>121/163<br>73%	$*_{MedF}$<br>54/90<br>59%	 16/54<br>30%	 27/85<br>32%	 22/62<br>35%	 7/24<br>29%
**Most common IAT**<br>- 1.<br>- 2.<br>- 3.<br>- 4.	$*_{MedM}$<br>nte (64%)<br>los (50%)<br>rit (46%)<br>iws (44%)	$*_{MedF}$<br>nte (44%)<br>los (39%)<br>iws (34%)<br>rit (32%)	nte (26%)<br>los (17%)<br>ign, sha (je 9%)	nte (24%)<br>los (14%)<br>ign, negprof (je 11%)	los (15%)<br>rit (14%)<br>nte, ign, negprof (je 11%)	los, rit (je 17%)<br>nte, ign (je 13%)
**IAT severity**<br>- not<br>- rather not<br>- rather<br>- very	$*_{MedM}$<br>20%<br>63%<br>13%<br>0%	$*_{MedF}$<br>43%<br>42%<br>15%<br>0%	 31%<br>63%<br>6%<br>0%	 58%<br>27%<br>15%<br>0%	 18%<br>41%<br>41%<br>0%	 57%<br>43%<br>0%<br>0%

The Table lists the descriptive and comparative statistical values. Not all participants stated their sex, therefore the sum of female and male students is less than the total number of participants.

Sex differences between participants from the same study program: * ≙ significant ($p<0.05$) + small effect size; ** ≙ significant + intermediate or large effect size; the indices indicate the groups for which a difference has been found: MedM = male medical students; MedF = female medical students; Abbreviations of IAT types: nte ≙ no teaching effect; los ≙ lack of supervision; iws ≙ insufficient work safety; rit ≙ requesting inadequate tasks; ign ≙ ignoring; sha ≙ shouting at, reprimanding; negprof ≙ negative remarks about professional topic

Attachment 2 to: Bormuth S, Ackermann H, Schulze J. *Inadequate treatment in interships: a comparison between medical and other students.* GMS J Med Educ. 2021;38(2):Doc45. DOI: 10.3205/001441
