# Supplementary material for: Inadequate treatment in internships: a comparison between medical and other students
Source: GMS J Med Educ. 2021 Feb 15;38(2):Doc45. doi: 10.3205/zma001441 (PMC7958909; doi:10.3205/zma001441)
Supplement: Figure S4 and table S9 [file JME-38-2-45-s-003.pdf]

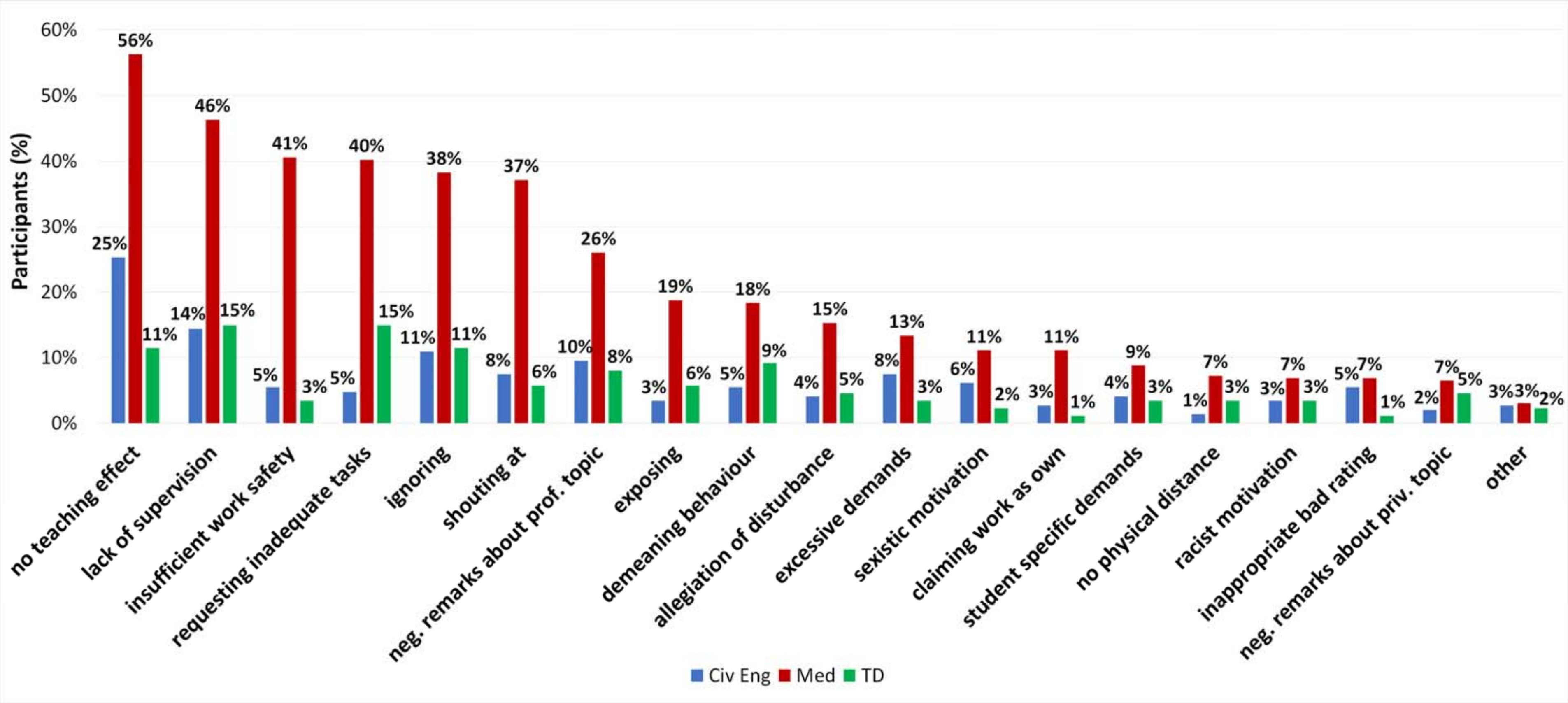

Figure S4: Comparison of IAT types in the three study programs. Values are based on all participants = 100%. Multiple answers result in a total > 100%.

Attachment 3 to: Bormuth S, Ackermann H, Schulze J. *Inadequate treatment in interships: a comparison between medical and other students.* GMS J Med Educ. 2021;38(2):Doc45. DOI: 10.3205/001441

Table S9: Subgroup analysis by sex; the table lists the parameters for the descriptive and comparative statistics.

	Medicine		Civil engineering		Teaching degree	
	**female**	**male**	**female**	**male**	**female**	**male**
**IATyes, qu. 5:**<br>- N_{IATyes} / N_{total}<br>- %	$^{*}_{MedM}$<br>121/163<br>73%	$^{*}_{MedF}$<br>54/90<br>59%	 16/54<br>30%	 27/85<br>32%	 22/62<br>35%	 7/24<br>29%
**Most frequent IAT:**<br>- 1.<br>- 2.<br>- 3.<br>- 4.	$^{*}_{MedM}$<br>nte (64%)<br>los (50%)<br>rit (46%)<br>iws (44%)	$^{*}_{MedF}$<br>nte (44%)<br>los (39%)<br>iws (34%)<br>rit (32%)	 nte (26%)<br>los (17%)<br>ign, sha (9% each)	 nte (24%)<br>los (14%)<br>ign, negprof (11% each)	 los (15%)<br>rit (14%)<br>nte, ign, negprof (11% each)	 los, rit (17% each)<br><br>nte, ign (13% each)
**Severity of IAT:**<br>- not<br>- rather not<br>- rather<br>- severe	$^{*}_{MedM}$<br>20%<br>63%<br>13%<br>0%	$^{*}_{MedF}$<br>43%<br>42%<br>15%<br>0%	 31%<br>63%<br>6%<br>0%	 58%<br>27%<br>15%<br>0%	 18%<br>41%<br>41%<br>0%	 57%<br>43%<br>0%<br>0%

Not all participants indicated their sex, therefore the total number is lower than the of participant total. For sex differences for students of a study program the indices indicate both significance and effect size: * ≙ statistically significant + low effect size; ** ≙ statistically significant + medium or large effect size; Index indicates the group differences: MedM = male medical students, MedF = female medical students. Abbreviations of IAT types: nte ≙ no teaching effect; los ≙ lack of supervision; iws ≙ insufficient work safety; rit ≙ requesting inadequate tasks; ign ≙ ignoring; sha ≙ shouting at, reprimanding; negprof ≙ negative remarks about professional topic
